# The Impact of a Low-Glucose Diet on Amniotic Fluid Index in Pregnant Women with Idiopathic Polyhydramnios: A Clinical Trial: Low-Glucose Diet and Amniotic Fluid Index in Pregnant Women

**DOI:** 10.31661/gmj.vi.3857

**Published:** 2025-06-29

**Authors:** Zahra Fardiazar, Rana Modabber, Hosein Azizi, Fatemeh Pourteymour Fard Tabrizi, Fatemeh Abbasalizadeh, Shamsi Abbasalizadeh

**Affiliations:** ^1^ Women’s Reproductive Health Research Center, Tabriz University of Medical Sciences, Tabriz, Iran; ^2^ Sarab Faculty of Medical Sciences, Sarab, Iran

**Keywords:** Polyhydramnios, Dietary Pattern, Pregnant, Amniotic Fluid

## Abstract

**Background:**

Polyhydramnios, defined as excessive amniotic fluid accumulation, increases the risk of maternal and fetal complications. In idiopathic cases, management options are limited. This prospective interventional study aimed to evaluate the effect of a low-glucose diet on the amniotic fluid index (AFI) in normoglycemic pregnant women with idiopathic polyhydramnios.

**Materials and Methods:**

A prospective interventional study was conducted involving 51 pregnant women between 24 to 35 weeks of gestation with idiopathic polyhydramnios at Al-Zahra Women’s Tertiary Referral University Hospital from 2024 to 2025. A low glucose dietary pattern was implemented by a nutrition specialist. AFI was measured at baseline, and two- and four-week’s post-intervention. To assess differences and changes in AFI across three visits paired T-tests and Repeated measures ANOVA were used.

**Results:**

The mean AFI levels at the initial visit, and at two and four weeks later, were 27.19, 22.41, and 19.5 cm, respectively. We noted a significant reduction in AFI at both the two- and four-week marks compared to the baseline (P= 0.001). The rate of decrease in AFI after two weeks (mean= 22.4, P= 0.001) was greater than that observed four weeks later (mean= 19.5; P= 0.004).

**Conclusion:**

The study demonstrated that reducing excessive oral sugar intake and modifying dietary habits resulted in a reduction of the amniotic fluid index (AFI) in pregnant women with idiopathic polyhydramnios.

## Introduction

Polyhydramnios is defined as an abnormal increase in the volume of amniotic fluid surrounding the fetus and may be associated with various maternal and fetal complications [1]. When not attributable to congenital anomalies of the central nervous or gastrointestinal systems, gestational diabetes, alloimmunization, fetal infections (e.g., cytomegalovirus or toxoplas-mosis), placental tumors, or multiple gestations, the condition is classified as idiopathic polyhydramnios. It complicates approximately 1-2% of pregnancies, with no identifiable cause found in about 60-70% of cases [2][3]. Idiopathic polyhydramnios is clinically significant due to its association with an increased risk of adverse outcomes, including preterm labor, abnormal fetal presentation, premature rupture of membranes, placental abruption, shoulder dystocia, cesarean delivery, and postpartum hemorrhage [4]. It also elevates the risk of neonatal complications such as low Apgar scores, intrauterine fetal demise, and perinatal mortality [5][6]. The condition is more common in women with diabetes mellitus, with approximately 15% of cases occurring in diabetic pregnancies [7]. The regulation of amniotic fluid volume is a dynamic process involving fetal urine production, fetal swallowing, and intramembranous and intravascular absorption [8]. Disruptions in these mechanisms—specifically impaired fetal swallowing or excessive fetal urine production—may result in polyhydramnios [9]. In maternal hyperglycemia, fetal hyperglycemia and subsequent polyuria can cause an abnormal increase in amniotic fluid volume [10]. Additionally, fetal glucosuria raises the osmolality of amniotic fluid, leading to fluid shifts into the amniotic cavity to maintain osmotic balance [11]. Although most data focus on hyperglycemia, it is physiologically plausible that maternal hypoglycemia could inversely impact fetal blood glucose levels, potentially reducing fetal urine output and consequently decreasing amniotic fluid volume. However, this scientific relationship between maternal hypoglycemia and reduction of AFI has been underexplored and forms only a small part of the current knowledge. Therefore, further investigation with robust clinical data is warranted to validate this potential association [12]. However, the direct relationship between maternal glucose intake and changes in amniotic fluid volume in normoglycemic pregnancies has not been firmly established. Most available studies focus on diabetic populations, and data regarding glucose intake’s effects in non-diabetic pregnancies remain scarce [13]. Amniotic fluid volume is typically assessed during routine ultrasound using subjective evaluation, measurement of the deepest vertical pocket (DVP), or calculation of the amniotic fluid index (AFI); polyhydramnios is generally diagnosed when the DVP exceeds 8 cm or the AFI is greater than 24-25 cm [14][15][16]. Currently available management strategies for polyhydramnios include invasive procedures such as amnioreduction or pharmacological therapies like indomethacin, both of which carry potential risks for the fetus and mother [17][18]. Given these limitations, there is growing interest in exploring non-invasive interventions, such as dietary modifications, to safely manage idiopathic polyhydramnios. Preliminary clinical observations suggest that reducing maternal sugar intake may contribute to a decrease in amniotic fluid volume; however, high-quality evidence supporting this approach remains limited [19][20]. Therefore, based on this clinical hypothesis and the need for safer alternatives, this prospective interventional study, registered as a clinical trial (registration ID: IRCT20230924028844N2), aimed to assess the speed and extent of amniotic fluid index reduction in normoglycemic pregnant women with idiopathic polyhydramnios following the implementation of a restricted-sugar diet. Maternal and fetal conditions were carefully monitored to ensure safety throughout the intervention. Nevertheless, the absence of a control group is acknowledged as a major limitation of the study, as a significant proportion of mild idiopathic polyhydramnios cases may resolve spontaneously without any intervention, making it challenging to attribute the observed changes solely to dietary modification [21][22].

## Materials and Methods

### Study population

A registered prospective interventional clinical trial was conducted among pregnant women with idiopathic polyhydramnios at Al-Zahra Women’s Tertiary Referral University Hospital from 2024 to 2025. The study evaluated the impact of a low-glucose diet on the amniotic fluid index (AFI) in pregnant women with idiopathic polyhydramnios.

According to the study by Khanduri S et al. [23], if the diet intervention could reduce 70% of the AFI, then considering a clinical difference of 0.2, a type I error of less than 5%, and a power of 80%, the sample size was estimated to be 50 subjects

**Figure-1:**
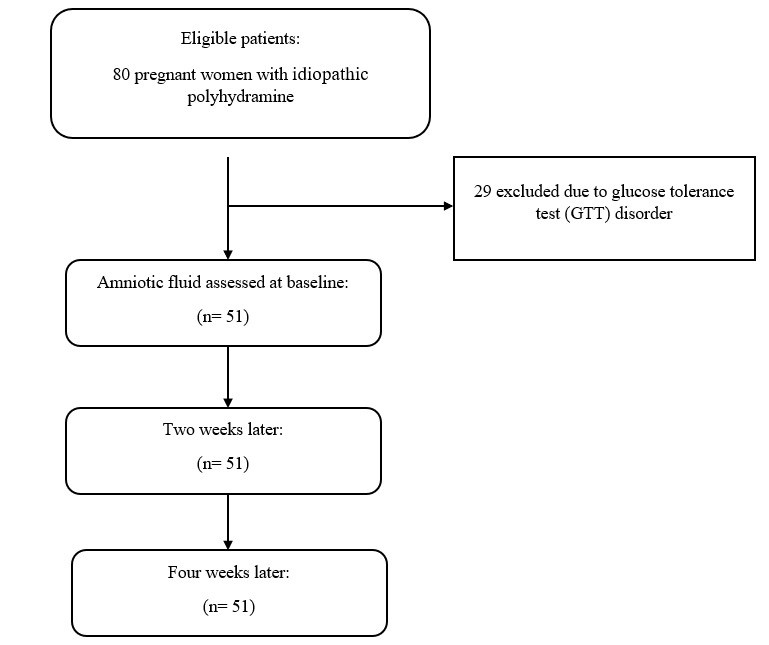
Figure 1. Flowchart of patient recruitment and measurements

Initially, 80 pregnant women with idiopathic polyhydramnios were screened. At baseline, fasting blood sugar (FBS) and glucose tolerance test (GTT) were assessed to exclude hyperglycemia. Twenty-nine cases were excluded due to abnormal GTT results. Finally, 51 eligible normoglycemic women with idiopathic polyhydramnios were included. AFI was measured at baseline, and again two and four weeks after dietary intervention (Figure-1).

Each participant served as her own control, and comparisons were made between baseline and post-intervention AFI measurements.Safety monitoring was performed throughout the study to ensure maternal and fetal well-being. It should be noted that no separate control group was included; thus, the possibility of spontaneous resolution of mild polyhydramnios is acknowledged as a study limitation.

### Eligibility

Inclusion criteria were pregnant women between 24 and 35 weeks of gestation with a singleton pregnancy. Participants were required to have an amniotic fluid index (AFI) at or above the 95th percentile for gestational age, or an absolute AFI measurement between 25 and 35 cm. They also needed to express willingness to adhere to the proposed dietary modifications for at least two consecutive weeks. Additionally, participants should not have had any medical indication for immediate delivery.

### Dietary interventions and Measurements

Pregnant women in the intervention group received a modified 14-day low-carbohydrate diet for managing gestational diabetes, designed by a dietitian. Participants were provided with dietary guidelines that were relevant to their needs, along with a specific meal plan. The nutritional guidelines for this diet were primarily based on established patterns for managing gestational diabetes. The diet plan was thoughtfully designed to promote small and frequent meals, ideally consisting of 4 to 6 servings throughout the day. It specifically aimed to limit simple carbohydrate intake to less than 10% of the total carbohydrates consumed daily. To achieve better nutritional balance, simple sugars were replaced with complex carbohydrates, such as whole grains, legumes, and vegetables, which provide extended energy release and essential nutrients. Furthermore, the diet emphasized the importance of increasing the intake of high-quality protein sources, including lean meats, fish, eggs, dairy products, legumes, and nuts, to support overall health and fetal development. Caloric intake was meticulously adjusted for each participant, taking into account their pre-pregnancy body mass index (BMI) and gestational age, ensuring that the dietary needs were tailored to promote optimal health for both the mother and the developing baby throughout the pregnancy.

The total calorie requirements for the participants were calculated by considering their pre-pregnancy BMI and gestational age, ensuring that the needs were tailored to their individual health statuses. Based on these calculations, the distribution of macronutrients was formulated according to a low-carbohydrate diet pattern, which was designed to have a macronutrient ratio of 18% protein, 50% carbohydrates, and 32% fat. This careful distribution aimed to support optimal health during pregnancy while managing weight gain. Subsequently, a detailed, personalized low-carbohydrate diet plan was created and provided to the subjects, including specific food choices and portion sizes to facilitate adherence and promote nutritional balance throughout their pregnancy. The diets were structured to include three main meals and three snacks, with a designated number of carbohydrate servings for each. No changes to the carbohydrate distribution were permitted during dietary therapy. Carbohydrate sources came from high-fiber, low-glycemic-index foods such as fruits, vegetables, whole grains, and pulses. The meals contained naturally occurring sugars, such as those found in fruits, but did not include any added sugars, preservatives, or artificial flavor enhancers. During the dietary interview, we employed graphic representations of food portions to help participants accurately estimate and report their food intake. These visual aids included images and diagrams of various servings, allowing for a clearer understanding of portion sizes and helping to facilitate more precise dietary assessments.

Women were asked to complete a 24-hour dietary recall and a three-day food diary to assess their typical diet. They were required to provide detailed information about their food consumption, including portion sizes and beverages. Adherence to the prescribed diet was evaluated through a telephone interview conducted at the midpoint of the study, which was the end of the first week.

### Statistical Analysis

Data analysis was conducted using SPSS version 19.0 (Chicago, IL, USA). The Shapiro-Wilk test assessed normality of continuous variables. Descriptive statistics summarized the data. Repeated measures ANOVA and paired t-tests compared AFI values at three time points: baseline (before intervention), two weeks, and four weeks post-intervention. A p-value <0.05 was considered statistically significant.

## Results

**Figure-2:**
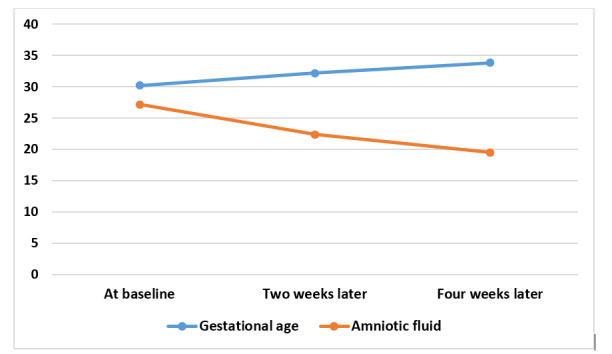
Figure 2. Plot for amniotic fluid index variations after dietary intervention by gestational age

**Table:** Table1. Demographic and medical history of the participants before intervention among pregnant women withpolyhydramine (n= 51)

**Variables**		**N**	**Percentage/SD**
Age (mean ± SD)		31.62	4.9
	Under diploma	9	17.6
Education	Diploma	29	56.9
	Academic	13	25.5
Occupation	Housewife	42	82.4
	employed	9	17.6
Blood pressure (drug used)	no	41	80.4
	yes	10	19.6
Complementary drug	no	-	-
	yes	51	100

**Table:** Table2. Baseline characteristics of the participants before intervention (n= 51)

**Variables**		**Mean**	**Standard Deviation (SD) **
Weight		68.45	8.5
BMI (Body mass index)		28.6	3.09
Gestational age at the baseline		30.2	1.8
Amniotic fluid index (AFI)		27.19	1.49
Fasting blood sugar (FBS),	mean(SD)	81.3	6.5
	70-80	24	47.1
FBS level (n, %)	80-85	25	49
	85-90	2	3.9
Blood Sugar (BS)	Mean (SD)	120.7	10.18
BS level (n, %)	100-120	27	52.9
	120-140	24	47.1

**Table:** Table3. Changes of AFI after dietary interventionamong pregnant women withpolyhydramine

**Follow-up times **	**Gestational age ** **(mean ± SD) **	**AFI** **(mean ± SD) **	**Pair T-test **	**F** (P-value) *
**At baseline **	30.21 (1.82)	27.19 (1.49)	Comparison of baseline with 2^nd^W (**P= 0.001**)	
**Two weeks later **	32.21 (1.82)	22.41 (2.86)		1951.5 **(P= 0.001**)
**Four weeks later **	33.78 (1.62)	19.5 (3.58)	Comparison of baseline with 4^nd^W (**P= 0.004**)	
**Treated ** **polyhydramine**	Under 25 (n, %)		43 (84.3%)	
**Macrosomia**	n; %		9 (17.6%)	

^*^Repeated measure ANOVA test **AFI:** amniotic fluid index

A total of 51 pregnant women with idiopathic polyhydramnios were analyzed, and AFI was evaluated at baseline, and 2 and 4 weeks later after dietary intervention. More details of the patient recruitment and measurements was demonstrated in (Figure-1). Table-1 shows the demographics and some medical history of the participants. Participants had an average age of 31.6 years. The housewives (82.4%) and diplomas educational level (57%) were the majority of those who participated. Out of the 51 pregnant women, 10 (19.6%) had high blood pressure and were receiving antihypertensive medications. Additionally, all participants received complementary drugs for pregnancy.

The baseline characteristics of the participants before the intervention can be seen in Table-2. The mean BMI (based on early pregnancy weight) was 28.6, respectively. The mean gestational age of the participants was 30.2 weeks at the study’s baseline. The averages for AFI, FBS, and BS were 27.19, 81.3, and 120.7, respectively.

Table-3 illustrates the changes in the AFI following dietary intervention among pregnant women with polyhydramnios, categorized by gestational age. The mean AFI levels at the initial visit, and two and four weeks later, were 27.19, 22.41, and 19.5, respectively. We observed a significant reduction in AFI at both the two- and four-week marks compared to the baseline (P= 0.001). The dietary intervention led to a significant overall decrease in the AFI (baseline, 2 and 4 weeks later, P= 0.001) and specifically after two weeks of intervention (P= 0.001). However, the rate of decrease in AFI after two weeks (mean= 22.4, P= 0.001) was greater than that observed four weeks later (mean= 19.5; P= 0.004).

Overall, out of 51 non-diabetic pregnant women who received dietary interventions, the amniotic fluid index was not changed (reduced) in eight patients. Two patients of these had abnormal neonate diagnosed after delivery.

Additional details regarding AFI changes by gestational age are provided in Figure-2.

Overall, 43 (84.3%) of polyhydramniosin pregnant women were improved after dietary intervention. Concerning adverse pregnancy outcomes, the rate of macrosomia was 9 (17.6%), and there were no any other complications such as umbilical cord prolapse or bleeding (Table-3).

Ethics approval and consent to participate

This study was approved by ethics committee of Tabriz University of Medical Sciences to number: IR.TBZMED.REC.1403.493. Informed consent was secured from all participants prior to the study.

## Discussion

This clinical trial demonstrated a significant role of a low-glucose diet on AFI in pregnant women with idiopathic polyhydramnios. In this study, each case served as its own control, and the impact of dietary intervention was compared before and after. Our results showed that applying dietary modification significantly reduced the amniotic fluid index in this group of women. The analysis results indicated that the rate of reduction was quite favorable between the baseline and two weeks post-intervention. However, during the period from two weeks to four weeks after the intervention, the rate of reduction in AFI was slower compared to the initial two weeks. The hypothesis that dietary glucose can directly influence amniotic fluid volume, even in normoglycemic mothers, is supported by previous studies indicating that glucose levels, even within normal ranges, can impact fetal urine production, which is a major contributor to amniotic fluid volume [24][25]. Thus, restricting glucose intake may reduce fetal osmotic diuresis, thereby lowering AFI even in the absence of overt maternal hyperglycemia.

In line with the current study, L. Tamayev et al. investigated the impact of a low-glucose, simple carbohydrate, high-vegetable, and fiber diet on the AFI among pregnant women with idiopathic polyhydramnios. Similar to our findings, they observed a significant reduction in AFI after two weeks of dietary intervention [26]. Regarding safety concerns, all participants in the intervention group were closely monitored with frequent prenatal visits and regular fetal assessments, including non-stress tests and ultrasound evaluations. No adverse maternal or fetal outcomes were observed, ensuring that the dietary intervention was both safe and effective throughout the study period. The main cause of idiopathic polyhydramnios in pregnant women is not fully understood, but high sugar intake and metabolic changes are among the suspected factors. A modified dietary pattern involving low free sugar, fruits, vegetables, and complex carbohydrates can potentially contribute to managing the condition. Although there was no external control group, each participant served as her own control by comparing AFI measurements before and after intervention, a design that minimizes inter-subject variability. Findings indicated that polyhydramnios is an independent risk factor for perinatal mortality. Those who were small for gestational age (SGA) and had polyhydramnios had the worst prognosis [27]. A registered diagnostic sonographer measured each woman’s four-quadrant AFI, following standardized procedures. In various studies across different countries including Germany, South Africa, the Netherlands, the United States, and Iceland, maternal sugar intake during pregnancy has been linked to adverse outcomes such as excessive weight gain and potential fluid imbalance, supporting the biological plausibility of our hypothesis [28][29]. Furthermore, the scientific basis for the relationship between maternal hypoglycemia and decreased AFI can be attributed to reduced fetal osmotic diuresis and subsequent lowering of amniotic fluid production [30]. It has been confirmed that excessive sugar intake during pregnancy is a nutritional and behavioral factor related to the development of gestational diabetes mellitus [28]. Despite the lack of direct studies linking high sugar intake to polyhydramnios, indirect evidence from studies on fetal growth, maternal hyperglycemia, and osmotic mechanisms supports this connection. Our findings are strengthened by supporting evidence from other studies showing that reductions in maternal glucose levels, even among non-diabetic pregnant women, can positively impact amniotic fluid regulation [24]. Moreover, the pattern of AFI reduction observed in our study—markedly significant during the first two weeks and slower thereafter—aligns with the expected physiological response to dietary changes, where an initial rapid effect is followed by a stabilization phase. This suggests a direct and timely impact of the intervention.

### Limitations

Our study found a positive impact of a low-glucose diet and dietary modification on AFI reduction in women with idiopathic polyhydramnios. However, there were some limitations. Although the study design minimized variability by using a before-after comparison within each subject, future research using randomized controlled trials could provide stronger evidence by eliminating potential time-dependent biases and controlling for external variables. Nevertheless, this clinical trial provides preliminary evidence supporting dietary intervention as a safe, non-invasive, and effective strategy for managing idiopathic polyhydramnios.

## Conclusion

The results of this clinical trial indicate that, despite a normal maternal glucose profile, avoiding excessive oral sugar intake and modifying the diet leads to a significant reduction in the amniotic fluid index (AFI) in pregnant women with idiopathic polyhydramnios. Furthermore, AFI is directly linked to high sugar consumption and gestational diabetes, suggesting its potential utility in the early prediction of gestational diabetes even prior to direct glucose measurement. Notably, the reduction in AFI was most pronounced in the gestational age range of 30 to 32 weeks, while the rate of decline slowed during subsequent weeks. However, the stabilization of AFI values in later pregnancy suggests that sustained long-term sugar restriction may help prevent complications associated with gestational diabetes and polyhydramnios.

## Conflict of Intrest

None.
